# Comparison of open and closed book test for admission in medical school

**DOI:** 10.15694/mep.2018.0000025.1

**Published:** 2018-01-25

**Authors:** Charline Cade, Jérémie Riou, Isabelle Richard, Catherine Passirani, Elisabeth Letertre, Anne-Victoire Fayolle

**Affiliations:** 1 General Practice Department; 2 MINT; 3 Faculté de Santé; 4 PluriPASS group

**Keywords:** Study skills, Medical education research, Evaluation, Medecine

## Abstract

This article was migrated. The article was marked as recommended.

**Introduction:** Students’ learning methods are highly influenced by the type of evaluation. Consequently, evaluation is a deciding step in the learning process. Multiple Choice Format Tests (MCQs) are almost exclusive in the admission to Health studies. *PluriPASS* suggests an educational reform of Health studies. This research aims at analyzing docimological features of Open-book Multiple Choice Format tests (OBT) compared to other usual tests used during the first year in Health studies curriculum (CC).

**Methods:** This educational research took place during *PluriPASS* year in Health studies curriculum at Angers University, during the academic period 2015-2016. The optional course « Disability and Health » (DH) was partly assessed by open-book tests with complex wording, requiring careful thought from students.

**Results:** Out of the 1161 students enrolled in first year curriculum, 190 have chosen the DH. For the students who followed the DH, the CC and OBT distributions are respectively a Skewness score at -0.11 and -0.12 and a Kurtosis score at -0.9 and -0.22. Bland Altman test or Deming method demonstrate a concordance between both method.

**Conclusion:** Distribution characteristics of OBT are satisfactory and allow to consider introducing this method with the objective of promoting reflection and depth learning.

## Introduction

Admission procedures have been considered by many authors as one of the most important step in the design of a medical curriculum ( Patterson & Al., 2016). Indeed in most higher education systems, the proportion of students that will complete medical studies after admission is very high and thus the admission procedure is crucial in shaping the working force for the health system. A large variety of procedures have been described and are currently used by medical schools in the world. ( Adam, Dowell, & Greatrix, 2011; Fayolle & Al., 2016; Groves, Gordon, & Ryan, 2007)

The admission process can take place at different points of the curriculum and it is roughly possible to distinguish undergraduate entry in medical schools, recruiting students immediately after secondary school and graduate entry curricula, recruiting students who have previously completed a bachelor course or equivalent. Some institutions offer both opportunities. ( James, Fergusson, Powis, Symonds, & Yates, 2008; Elliott & Epstein, 2005)

French regulations allow all students having completed secondary education to apply to university in most fields, including health sciences ( Arrêté du 28 octobre 2009). Students are admitted in a multidisciplinary first year program designed for large numbers of students and compulsory to apply as a second year student for medicine, pharmacy, dentistry and midwifery studies. Therefore the real bottleneck for admission to health studies is the end of first year competitive exam, which can be compared to the admission procedure of other higher education systems.

The processes used for screening, and finally selecting medical students vary from one institution to another. Most systems include the assessment of academic achievements, either by some measurement of secondary education achievements (such as grade point averages, marks for final secondary education exams, etc...), or by specific entrance exams assessing previously acquired knowledge. Some systems include tests specifically designed for recruiting medical students, sometimes on a nationwide basis, such as the MCAT (Medical college admission test) ( Pau & Al., 2013) or the UKCAT (United Kingdom Clinical aptitude test) ( Reiter & Eva, 2005). In many medical schools the application procedure includes a written essay of the motivations, support letters, and attention to prior activities in the field of health or community support activities. Some medical schools include an interview, usually for a subset of selected students. Several interview patterns have been described among which the Multiple Mini Interviews developed by Macmaster University ( Eva, Rosenfeld, Reiter, & Norman, 2004) and widely applied in Canadian medical schools ( Pau & Al., 2013).

Designing the admission procedure involves several steps and several stakeholders.

The first step determines the applicant’s characteristics that are considered as relevant for admission. The trend is toward a shift from assessment of purely academic characteristics to a more competency-based assessment, including for instance communication skills or ethical reasoning ( Reiter & Eva, 2005). The second step is designing tests or procedures that accurately assess these characteristics. Since the number of applicants is usually much higher than the number of available positions, the ability of the procedure to discriminate between students appears more determinant than the ability to set a minimal level. In other words many students applying for medical school will perform fairly well on many procedures and the challenge is to distinguish very good candidates from good candidates.

The stakeholders appear to have different priorities in designing the admission procedures ^9^. The health system, and for instance the representatives of the future patients have expressed their concern that the admission procedure should ensure that the students have the relevant communication and ethic reasoning skills. The students are extremely sensitive to the fairness of the procedure; and the universities need to control the overall organization costs ( Hissbach, Sehner, Harendza, & Hampe, 2014).

In France, the first year competitive exam consists exclusively in written exams, including multiple choice questions for a set of basic science subjects and an essay for social sciences. In all French universities these exams are closed book exams. The multiple choice questions have usually fairly short stems and assess mainly basic recall. These procedures have been accused of selecting “memory freaks”, and being irrelevant when it comes to assessing the ability to deal with uncertainty ( Edutopia,2017). Despite a number of criticisms these procedures have been maintained because of their easy and cheap implementation, and because they are well accepted by students. Students associations consider them to be fair even if they might admit that they are incompletely relevant.

The French ministry of higher education has issued in 2013 a call for proposal and some universities have been selected to implement different admission procedures. The admission program (called PluriPASS) implemented at the university of Angers has been completely redesigned and consists in a core program and elective self selected modules. These modules represent only a small proportion of the final mark and have been used to develop and test different learning strategies and different assessment procedures. One of these modules (Disability and Health (DH) module) consists of a 20 hours online content presenting concepts and issues related to disability. The assessment of this module is an open book exam of 15 multiple choice questions with long stems, completed in one hour.

Open book tests (OBT) have been widely described and studied ( Feller,1994). Open book exams could favor deep learning strategies and could be a method to assess how the student uses available knowledge rather than memorizing large amounts of data. This could be particularly relevant in a world of growing available information. Therefore open book multiple choice questions could be a strategy that combines low cost, reproducibility and “fairness” of multiple choice questions as well as the ability to investigate deeper cognitive strategies. OBT have also showed a trend towards better results for all or most students ( Feller,1994; Tarrant, Ware, & Mohammed, 2009; Stalnaker & Stalnaker,1934; Heijne-Penninga, Kuks, Hofman, & Cohen-Schotanus, 2010;). This could be a limit in using OBT for admission procedures if the distribution of the marks is such that distinguishing excellent from good students on this basis becomes difficult.

The objective of this study is to analyze the results on an open book test in a first year student population, and compare them to closed book test (CBT) results.

## Methods

### Population and Method

The study concerns the population of students enrolled in the first year program during the 2015-2016 academic year at the University of Angers (France) who selected the “Disability and Health” module (DH Module).

The first year program included a core curriculum of 12 subjects. Each subject was assessed by a CBT multiple choice question (MCQ) exam and for two subjects in the field of social sciences by MCQs and a short essay. Students obtained a mark which was the sum of the 12 marks (maximum 1200 points).

Each student also selected three elective modules. These modules were assessed by a range of methods. The DH module assessment included a one hour open book exam. The results obtained for the elective modules were considered on a fail/pass basis and a “pass” result provided an additional 20 bonus points.

The total mark (including the sum of the core curriculum marks and the possible additional elective bonuses) was used to rank the students for admission to the second year of medical studies. Based on this result the 63 students with the highest marks were admitted. The 119 following students were invited for additional interviews. 64 of these students were admitted after the interviews.

Age, gender, core curriculum mark and final admission status were obtained for all students.

For the sub-population of “DH module students” the marks for the OBT was extracted.

### Data processing

The characteristics of the sub-population of DH students were compared to the overall population by student test, or test and Fisher’s exact test, as appropriate.

The core-curriculum marks were expressed as a fraction of 20 (sum of the 12 modules (max 1200) /60), which is the usual expression of marks in the French academic system.

The distribution of core-curriculum marks, and DH module book test were established. Each distribution was described by the Skewness and Kurtosis coefficients ( Groeneveld & Meeden, 1984). The Skewness coefficient describes how symmetrical the distribution is and the Kurtosis coefficient how sharp (or flat). The value of both coefficients is 0 for a normal distribution. This analysis was performed to obtain data as to the ability of each method to discriminate and rank students. For instance, a very high Kurtosis coefficient would mean that the distribution is very sharp and that many students obtain the same mark, which is inappropriate if the aim is to rank students.

The correlations between the marks obtained by a given student for the core curriculum and for OBT of the DH module were analyzed by the graphical method of Bland and Altman ( Martin & Altman, 1986). This method is used to determine whether one measure is systematically biased against another and whether both methods are consistent, by determining the percentage of differences within an interval of two standard deviations. Finally, considering that these marks are used to decide admission, we defined the number of students who ranked in the top 20% for each method.

## Results

### Population

**Figure 1.**
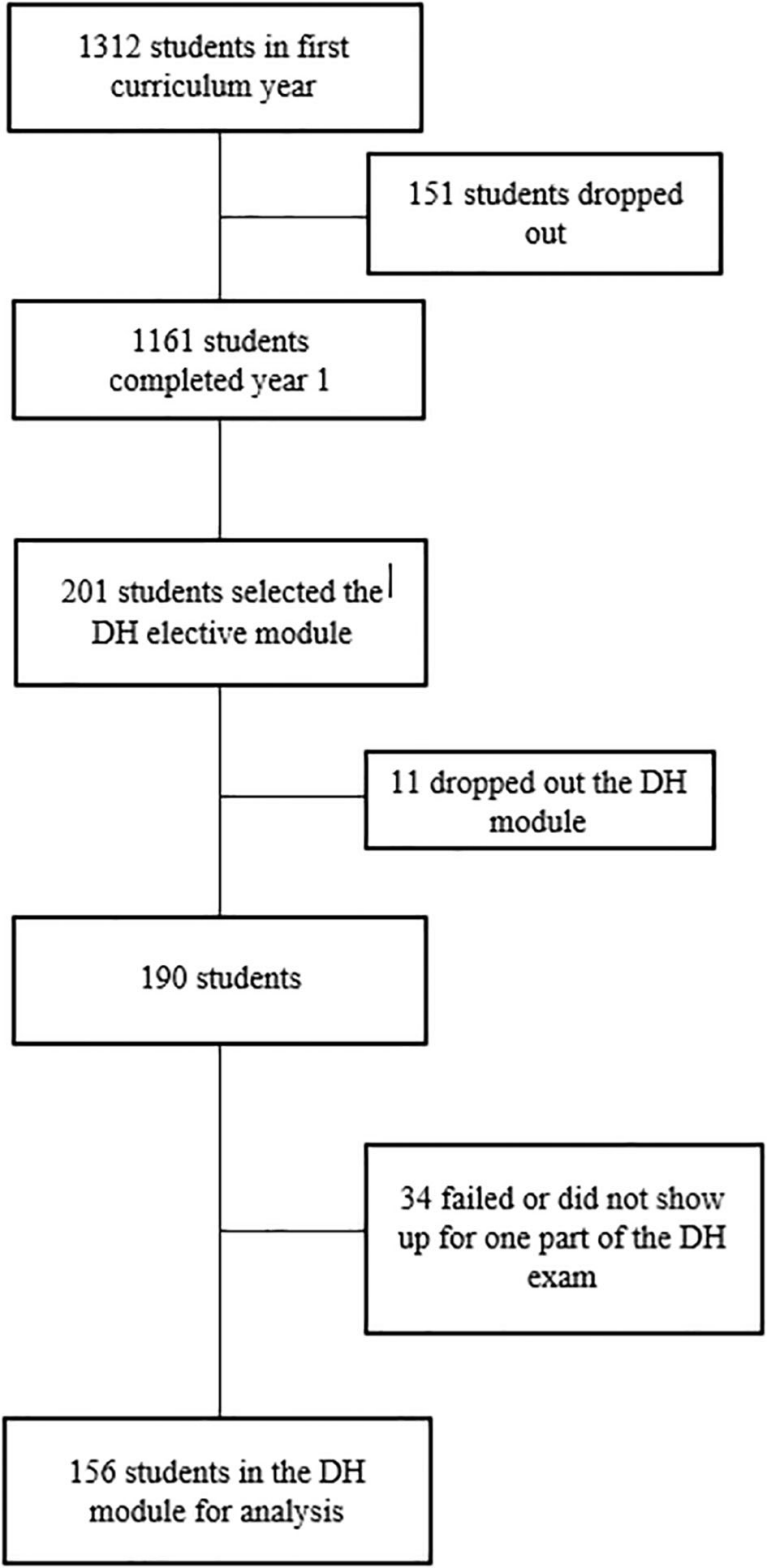
Students flow chart.

1312 students entered in September 2015 the first curriculum year. 151 dropped out during the first month and did not show up for the first exam. ( Figure 1)

DH: “Disability and Health” optional course

201 students selected the DH elective module; compared to the overall population these students are more often women (88% versus 70.5%, p <0.05) and do not differ on other characteristics such as their achievements for the core-curriculum modules or their final admission status. ( Table 1)

**Table 1.**
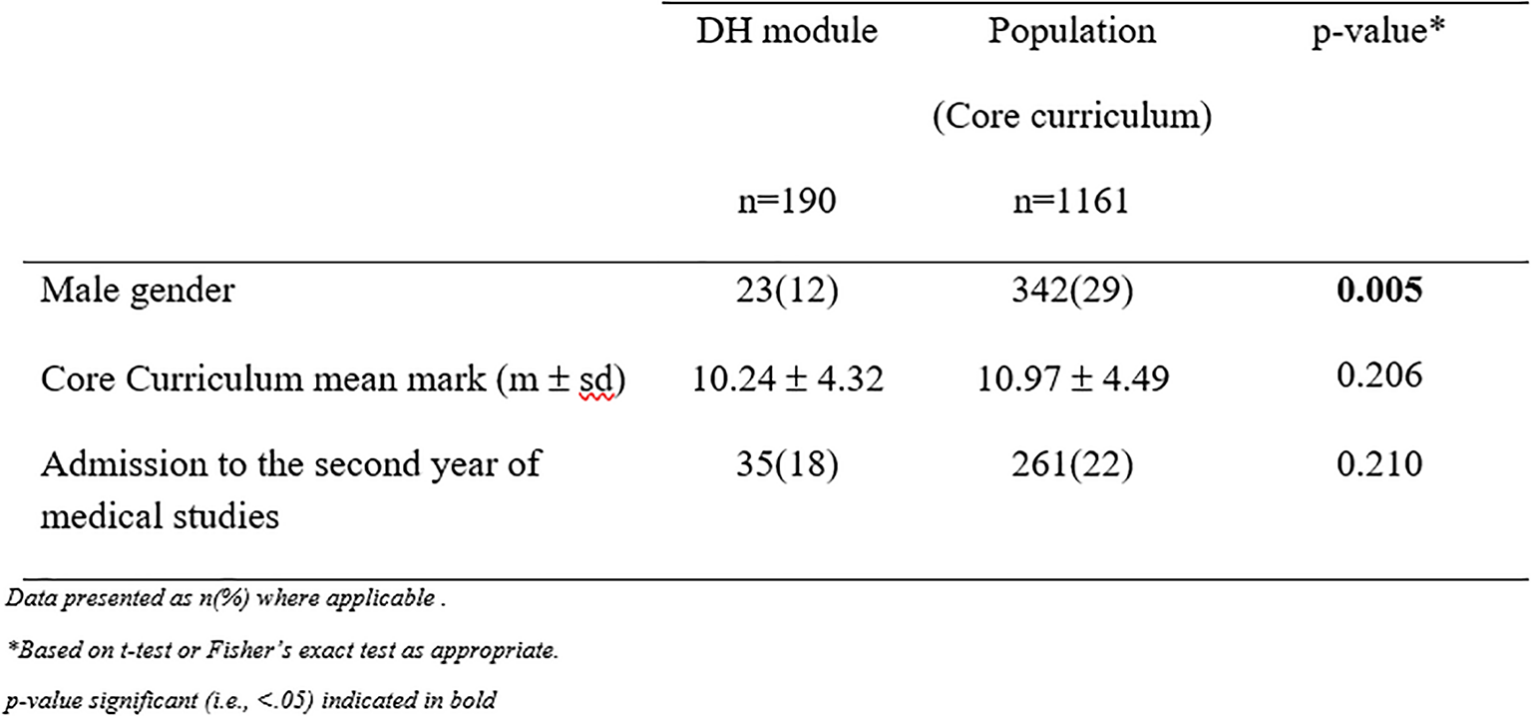
Students’ characteristics in PluriPASS. Comparison of the sub-population of DH students to the overall population.

Thirty-four students having selected the DH module dropped out and did not show up for the DH exam. These students also achieved very poor results on the core curriculum modules as they did not show up for some or all exams and were removed from further analysis.

### Marks distribution

**Figure 2.**
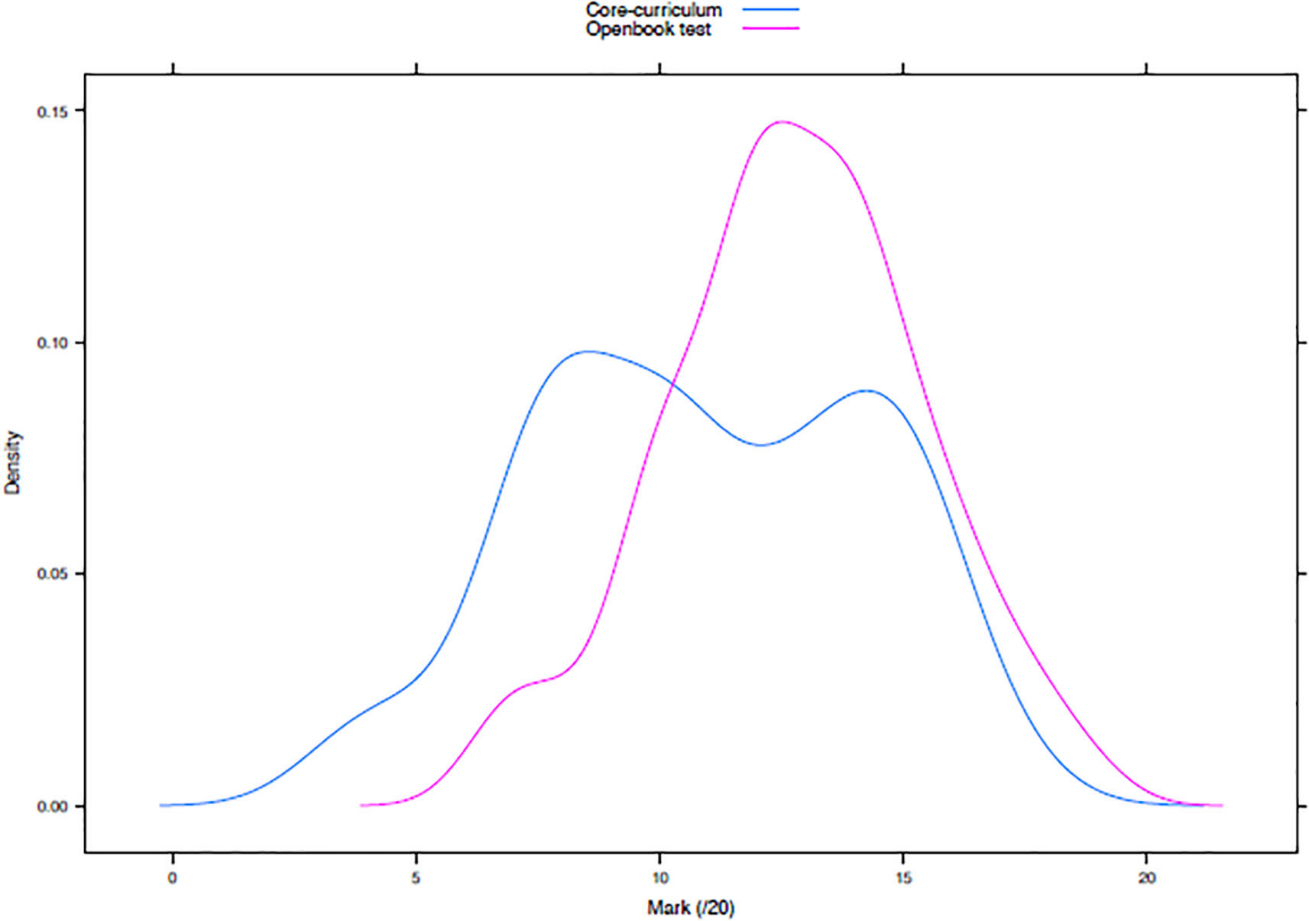
Superposition of distribution curves: core-curriculum (blue) and Open-book Test (pink).

The distribution of the marks was analyzed for the core-curriculum mark and for OBT and is presented in the ( Figure 2). All marks are expressed as a fraction of 20.

The mean (10.88 in core curriculum vs. 12.77 in OBT), median (10.55 vs. 12.82), variance (11.89 vs. 7.02), skewness (-0.11 vs. -0.12) and kurtosis coefficients (-0.9 vs. -0.22) are presented in Table 2.

**Table 2.**
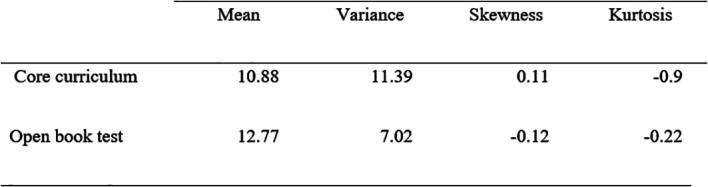
Marks characteristics of core curriculum and Open book test

The mean mark is significantly higher for the OBT than the average core-curriculum mark (12.77 vs. 10.88, p<0.05 ).

### Homogeneity of methods

**Figure 3.**
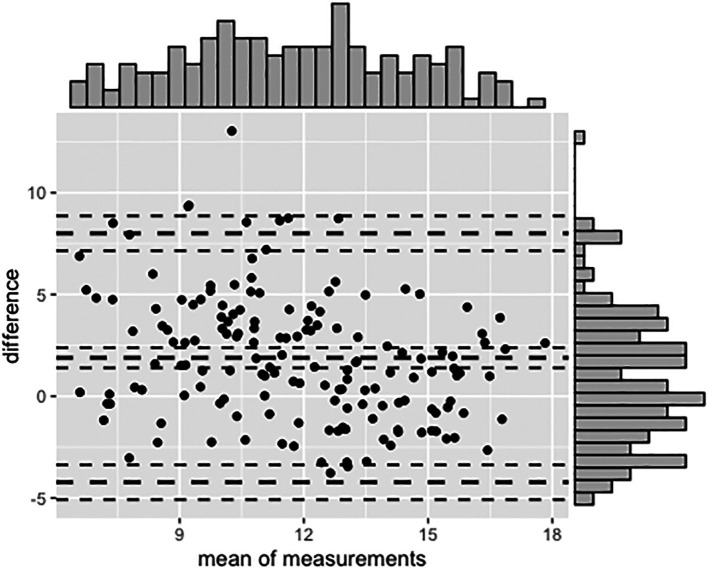
Bland Altman analysis to compare the homogeneity of the Open Book Test and Core Curriculum marks.

The Bland Altman analysis was applied to compare the homogeneity of the OBT and core curriculum marks (CC) ( Figure 3). As previously stated the average observed bias is 1.89. The graph shows that few points stand outside the amenity limits set at two standard deviations around the observed systematic difference.

Finally we determined which students belonged to the top 20 % for both methods and determined the conditional probability of belonging to the top 20 % on the OBT knowing that a student belongs to the top 20 % of the core curriculum and vice-versa. This shows that among the top 20 % for the CC mark, 50 % also belong to the top 20 % for the OBT (and 50% do not).

## Discussion

Our results show that students obtain higher marks on OBT of the elective module than on the CBT of the core curriculum. The distribution of OBT marks remains fairly symmetrical as attested by the skewness coefficient close to zero. The distribution is sharper than for the CBT but remains flatter than a normal distribution. 10 % of the students belong to the top 20 % for both methods and 10 % belong to the top 20 % for one method but not the other. The OBT tends to be “easier” which is an issue to take a pass/fail decision and slightly less discriminant which is an issue if the goal is to rank students. Both methods appear homogeneous as attested by the small number of differences greater than two standard deviations.

The main strength of this study is the fairly large sample of students who were selected for their participation in the study. A number of bias do exist, among which the fact that students who took the DH module were more frequently women in comparison to the core curriculum population, and the fact that the mark was not directly used to rank the students but to provide a bonus on a pass/fail basis.

One of the objectives of our institution in implementing this new first year program was to try to encourage deep learning and assess students on other competencies than the ability to memorize large amounts of data. Multiple mini interviews were for the first time in France introduced in first year health students’ selection method. Cost and feasibility issues made the choice of multiple choice questions, with automatic marking unavoidable.

A number of methods have been described to design multiple choice questions ( Heijne-Penninga, Kuks, Hofman, & Cohen-Schotanus, 2008; Moore & Jensen, 2007) which may assess strategic thinking reasoning and decision making; multiple choice questions typically include a context, a question, and a set of 4 or more possible answers. They are considered as rich context questions if the context includes several data such as a text, possibly tables, graphs or figures, that need to be manipulated to answer the question. Typically multiple choice questions developed for the assessment of clinical reasoning such as the USMLE (United States Medical Licensing Examination) tests ( Green, Angoff, & Encandela, 2016) are rich context questions. Another strategy is to have sequential questions and make it impossible for the student to go back to the previous step. This allows to test sequential reasoning for instance clinical reasoning by adding information. This is much easier with computer-based assessments and has been used on a large scale in France for the exam giving access to the residency programs. One of the limits in implementing rich context questions is that they require important expertise for the question writing. The instructions for authors of the USMLE test are complex. ( Green, Angoff, & Encandela, 2016)

Open book exams have been described in multiple settings as a strategy to assess how a student uses information rather than whether he has memorized information. Brightwell and al. describe five characteristics of OBT : they require skills, induce creative use of course content, increase self-feedback on competences, decrease stress during the examination and, finally, allow the students themselves to regulate the content of the studies ( Brightwell, Daniel, & Stewart, 2004). This encourages reflection rather than memorization in a relaxed environment, which encourages students to study appropriate subjects ( Bloom & Al., 1971; Eilertsen & Valdermo, 2000). In a world in which information is easily available on any mobile device, the “future of education” and therefore of assessment should, according to Ioannidou, focus on reasoning, conceptualization and problem solving rather than “storage” capabilities ( Ioannidou,1997). This idea is long-standing since, as early as 1934, Stalnaker ( Stalnaker & Stalnaker, 1934) suggested that open-book evaluation should be the norm for the assessment of higher education because it places less emphasis on memorization and encourages deeper commitment from students. They are encouraged to structure their own basic knowledge. Feller argued in 1994 that open book evaluation is superior to closed book reading because it is more realistic and similar to the “real life” situations that students may encounter outside the academic world ( Feller, 1994). Different methods for OBT have been described and some authors describe intermediate strategies with the use during the exams of “cheat sheets” consisting of a single or double page of information prepared by the student. ( Erbe,2007; Whitley & Bernard, 1996). Such strategies could favor deep learning for the student being motivated to organize the learning material ( Dickson & Bauer, 2008).

Changing assessment methods is difficult and was a very controversial issue in implementing this experimental first year program. Our institution had limited experience of OBT and most teachers were not experienced in writing MCQs with rich contexts. Therefore the elective modules were used to test innovative assessment strategies and define whether their properties could allow using them in the core curriculum.

The results of the students were better on OBT than on the core curriculum, despite the fact that the “bonus” system did not encourage students to get marks above the pass threshold. This has been previously described for OBT ( Chan & Mui, 2004; Skidmore & Aagaard, 2004). Authors have shown that this is not due to more efficient learning method when preparing for OBT ( Dickson & Bauer, 2008), and that OBT is not relevant if the objective is to assess the correct memorization of core knowledge, can lead students to reduce their workload and can decrease long-term retention.

The absolute measure is important if the objective of the assessment is to set the minimal level that students should achieve (for instance to get a “pass” result). In such contexts the pass threshold could be set at a higher level for OBT than for CBT.

The distribution of the marks for the OBT was not very different from the distribution for other tests, and the Kurtosis coefficient, which assesses how flat the curve is was lower than for a normal distribution. This parameter is crucial if the objective is to rank students and therefore spread the distribution. The intuitive concern that OBT could lead to all students achieving high marks making it impossible to rank them effectively was not confirmed and we believe that this preliminary result makes it safe to introduce some proportion of open book testing in the core curriculum.

In admission procedures, one of the key issues is whether the student is within the top 20 % who are offered admission. Comparing the core curriculum marks and OBT, 10 % of the students fall in the top 20 % in both cases, and 10 % for one method but not for the other. This could indicate that the abilities assessed by both methods are indeed different.

Open book multiple choice questions are more frequently rich context questions and this was the case in our study. Indeed the teacher is fully aware that the students will bring their notes and anticipates that testing purely factual knowledge is not relevant, and therefore tends to write complex questions requiring the students reasoning. This can also be done for close book tests if the teachers are clearly instructed to do so. One interpretation of our results could be that the open book rich context questions did indeed assess reasoning, more or differently from the questions of the core curriculum, but it is not possible to determine if this is due to the open book testing or to the rich context. Open book test appears in fact as an institutional backdoor to motivate teacher to write rich context questions. Further work could differentiate rich context open book tests and rich context closed book tests.

## Notes On Contributors

**Authors’ names:** Charline Cade ^1^; Jérémie Riou ^2^; Isabelle Richard ^3^; Catherine Passirani ^4^; Elisabeth Letertre ^4^; Anne-Victoire Fayolle ^1^

Affiliations: (department, university, city, country)

^1^General Practice Department, University of Angers, France

^2^MINT, UNIV Angers, INSERM 1066, CNRS 6021, Bretagne Loire University, IBS- CHU, Angers, France

^3^Faculté de Santé, University of Angers, France

^4^PluriPASS group, Faculté de Santé, University of Angers, France

Authors’ contributions: All authors read and approved the final manuscript

## Appendices

### List of abbreviations

CBT: Closed book test

CC: Health studies curriculum

DH: “Disability and Health” optional course

MCAT: Medical college admission test

MCQ: Multiple choice format test

OBT: Open-book Multiple choice format test

UKCAT: United Kingdom clinical aptitude test

USMLE: United States Medical Licensing Examination

### Declarations

Ethics approval and consent to participate: Not applicable

Consent for publication: Not applicable

Availability of data and material: Not applicable

Competing interests: None

Funding: None
