# Supplementary material for: Comparison of open and closed book test for admission in medical school
Source: MedEdPublish (2016). 2018 Jan 25;7:25. [Version 1] doi: 10.15694/mep.2018.0000025.1 (PMC10712008; doi:10.15694/mep.2018.0000025.1)
Supplement: Peer review history

# Reviewer report: Reviewer response for version 1

**DOI:** 10.21956/mep.19495.r28808

**Published:** 2019-05-01

**Authors:** Ken Masters

**Affiliations:** 1 Sultan Qaboos University

This review has been migrated. The reviewer awarded 3 stars out of 5 A reasonably interesting paper dealing with a comparison of open-book testing as opposed to closed-book testing for entrance tests to medical schools. The authors have properly identified the central problem and the contextual literature background, have performed a detailed analysis of the data (with a strong sample size), and then have related their Results back to the literature in the Discussion.The paper has many strengths, but, unfortunately, is undermined by some careless work in the presentation.Some things to consider:• It is a pity that the authors elected to not have a separate Conclusion, and did not use the “Take Home Messages” option of the journal. In this paper, a large amount of information has been given, and the Discussion is very detailed. This is a strength of the paper. An associated weakness, however, is that many readers can become overwhelmed, and not see the overall impact of the study. A succinct Conclusion and bullet-point “Take Home Messages” helps significantly to clarify the central messages of the paper. • Quite a few small errors, such as errors of language, upper-case and lower-case inconsistency, lack of commas where they should be, typographical errors, incorrect word order, etc. These really should have been fixed before submission, as they serve only to irritate the reader.• The conclusion of the Abstract is really difficult to follow, and some greater care should have been taken on making sure that this is very clear.So, a useful study, but the authors really should have taken greater care in the presentation of their material.

Reviewer Expertise:

NA

No decision status is available

# Reviewer report: Reviewer response for version 1

**DOI:** 10.21956/mep.19495.r28807

**Published:** 2018-01-25

**Authors:** P Ravi Shankar

**Affiliations:** 1 American International Medical University

This review has been migrated. The reviewer awarded 4 stars out of 5 I enjoyed reading this article about an open book exam as part of the admission process in a French University. The authors also provide a good overview of student admission to medicine. I also obtained an overview of the admission process to the French system of medical education. Open book tests can encourage critical thinking and application of information by the students. The authors describe how in the modern era technology has made it easier to access information. Hence, learning to interpret and apply information may be a very important skill. Modern doctors in practice have ready access to electronic resources which can quickly retrieve information. The authors can provide ‘Take Home Messages’ toward the end of their manuscript. A brief description about the University of Angers and the medical school will be helpful to international readers. The standard of written English in the manuscript is good, though corrections/modifications can be carried out in a few places. Admission tests using knowledge-based multiple choice questions are widely used in many institutions and countries mainly due to ease of standardization, perceived objectivity and the need for lesser resources. Many institutions are increasingly using other methods to evaluate applicants. This paper will be of interest to medical educators and especially to those with a special interest in student admissions.

Reviewer Expertise:

NA

No decision status is available
